# Effectiveness of the Australian MATES in Construction Suicide Prevention Program: a systematic review

**DOI:** 10.1093/heapro/daad082

**Published:** 2023-08-30

**Authors:** Jorgen Gullestrup, Tania King, Samantha L Thomas, Anthony D LaMontagne

**Affiliations:** Institute for Health Transformation, School of Health and Social Development, Deakin University, Geelong, Australia; School of Population and Global Health, University of Melbourne, Melbourne, Victoria, Australia; Institute for Health Transformation, School of Health and Social Development, Deakin University, Geelong, Australia; Institute for Health Transformation, School of Health and Social Development, Deakin University, Geelong, Australia

**Keywords:** suicide prevention, mental health, workplace, engaging men, public health

## Abstract

Suicide is a major public health issue globally. The World Health Organization has called for nations to create comprehensive national suicide prevention strategies including multisectoral collaboration, awareness raising, advocacy and capacity building. The workplace provides opportunity and structure for suicide prevention programs. However, many of these programs are poorly documented and evaluated. The MATES in Construction (MATES) program is a multimodal workplace-based suicide prevention program designed for and by the construction industry. This systematic review examined the available evidence for the effectiveness of the MATES program and is reported according to PRISMA guidelines. A literature search resulted in the inclusion of 12 peer-reviewed articles published between January 2010 and February 2023 containing primary data of evaluations of MATES. There was evidence of the effectiveness of the MATES program in improving mental health and suicide prevention literacy, helping intentions and reducing stigma. The results highlighted the importance of worker-to-worker peer approaches with workers consistently stating that supervisors were the least trusted resources for mental health and suicide concerns. Favourable results were found in relation to reduced suicide risk in the construction industry. The evidence base for MATES is limited in terms of causal inference with very few controlled evaluations and no experimental studies having been conducted to date. Improved understanding of how the program motivates volunteers, their experiences and research on the longer-term impacts of the program on the industry is required.

**Contribution to Health Promotion**

- The study found some support for the MATES ‘Outrage, Hope, Action’ Model of engaging men in collective action preventing suicide.
- MATES demonstrates the efficacy for a focus on help offering over help seeking in male suicide prevention.
- The findings from this study have wider applications in providing health promotion to male-dominated populations.

## INTRODUCTION

The World Health Organization (WHO) has stated that suicide and suicide behaviours are a critical public health issue (WHO, 2021a). Globally more than 800,000 people die by suicide each year with suicide amongst the leading causes of death across Europe, Australia and North and Latin America (Naghavi, 2019). In developed countries, suicide rates are generally 3−4 times higher for men than for women (Chang *et al.*, 2019) and it is estimated that for every death by suicide, 10−20 individuals attempt suicide and 17% of all suicide attempts cause permanent disability (WHO, 2009).

In 2021, the WHO developed a guide for national strategies on suicide prevention, including the core pillars of multisectoral collaboration, awareness raising, advocacy and capacity building, as well as the need for scalable prevention and health promotion initiatives (WHO, 2021b). Rogers has described diffusion as the process through which an innovation is communicated through certain channels over time among members of a social system (Rogers, 2002). A significant barrier to the diffusion of preventive health innovations is that the reward for adopting preventative innovations is delayed over time and the rewards are often intangible — something that is especially true for a relatively rare (but catastrophic) health event such as suicide. There are also unique complexities with scaling up some initiatives for different population subgroups. While programs that are more likely to be effective in reaching men consider both masculine norms *and* socio-cultural determinants, engage men through ‘doing’, use language acceptable to men, and change norms through peer engagement (Oliffe *et al.*, 2020), very few programs have been able to reach a large male audience in suicide prevention.

Workplaces are important venues for mental health and suicide prevention as they are practical settings, particularly for male-dominated groups that otherwise can be difficult to reach (Seaton *et al.*, 2019), and because employers have a duty of care to mitigate psychosocial hazards in the workplace (Potter *et al.*, 2019). Construction workers have been repeatedly identified as being at high risk of suicide, with global suicide rates amongst construction workers on average 25% higher than comparison groups (Tyler *et al.*, 2022c). The Australian construction industry employs more than 10% of the Australian workforce and is a significant contributor to the economy (ABS, 2020). Construction is highly male dominated, with 88% of workers identifying as male against an overall workforce average of 53% (ABS, 2020). Consistent with global studies, Australian construction workers have been found to have rates of suicide more than twice that of other employed men (Maheen *et al.*, 2022). Workers in the construction industry are also significantly more likely to experience psychosocial job adversity and exhibit traditional masculine norms (Tyler *et al.*, 2022a). A study of construction apprentices found that 29% of participating apprentices reported suicide ideation in the previous year (Ross *et al.*, 2022) with 31% reporting exposure to bullying in the previous 6 months, 13% reporting elevated psychological distress and 30% reporting reduced well-being (Ross *et al.*, 2021). While suicide and suicide ideation is multifaceted and will often be impacted by personal-, industry- and work-related risk factors (Tyler *et al.*, 2022b), many of the work-related risk factors for suicide have also been associated with higher risk of physical injuries at work in the construction industry (Alqahtani *et al.*, 2022). Despite the importance of workplaces in mental health and suicide risk, very few workplace-focussed mental health or suicide prevention interventions have been documented, with few evaluations of existing initiatives published in the peer reviewed or grey literature (Milner *et al.*, 2015; Seaton *et al.*, 2019; Greiner *et al.*, 2022).

The Australian program MATES in Construction, hereinafter MATES, is an example of a multisectoral collaboration raising awareness and building resilience in relation to suicide in construction workers (WHO, 2021b). MATES is a comprehensive and multimodal industry-based suicide prevention program (Martin and Gullestrup, 2014; MATES in Construction, 2020a; Neis and Neil, 2020), and is one of the few well-documented and evaluated workplace-based suicide prevention programs globally (Milner *et al.*, 2015). For a description of the program see Supplementary Material A. It is an integrated industry intervention program that raises awareness of suicide as a preventable problem, builds stronger and more resilient workers, connects workers to the most suitable available help, and finally, supports and partners with researchers to inform industry on best practice (MATES in Construction, 2020a). Originally designed as ‘Men Actively Talking to Each other on Sites’, MATES was set up in Queensland in 2007 in response to the documentation of elevated suicide risk in the sector compared with other Australian men (AISRAP, 2006; Heller *et al.*, 2007; Neis and Neil, 2020).

MATES has trained more than 237,359 workers in General Awareness Training (GAT) and supports a network of 21,888 ‘Connectors’, workers volunteering to be the connection point between workers in distress and support resources and 2889 ‘ASIST workers’, workers volunteering to be a support resource for colleagues in distress across the Australian construction industry (MATES in Construction, 2020b). MATES is noteworthy due to the program’s successful diffusion amongst construction workers leading to wide dissemination in target areas (Rogers, 2002; Oliffe *et al.*, 2020). MATES uses an ‘Outrage, Hope, Action’ Model (LaMontagne and Shann, 2020) to engage and motivate workers. The core objective of MATES is to increase awareness and engage workers collectively in suicide prevention (Martin and Gullestrup, 2014; Neis and Neil, 2020). MATES has inspired other workplace mental health and suicide prevention programs such as the ‘Blue Hats’ program in Australia (Beavis, 2019), ‘Mates in Mind’ in England (IOSH Magazine, 2017) and has also been extended to the mining and energy industries and the New Zealand construction industry (Little, 2019), with significant interest from several other male-dominated industries in Australia.

The MATES program logic model describes how program outputs are expected to generate program outcomes (Table 1) including improved mental health and reduced suicidality (LaMontagne and Shann, 2020). MATES is a program rolled out organically and continuously over time. In this context short-, medium- and long-term outcomes are to be understood as referring to the order of the outcomes more than a timeframe (LaMontagne and Shann, 2020). The following systematic review aimed to document available published evidence about the MATES program and assesses the evidence for overall program effectiveness.

**Table 1:** List of MATES program logic model outcomes

Outcomes		
Short-term	Medium-term	Long-term
MATES staff feel supported and satisfied. **Increased mental health and suicide literacy and decreased public stigma.** **Workers and volunteers play an active role in better mental health and suicide prevention, driving local activities.** **Workers and/or family members obtain support from MATES or volunteers.** An active coalition of representatives from industry, families, MATES staff, policy makers, other industry leaders & academics is formed with a clear mission and purpose and evidence of a commitment to meaningful activity.	**Industry see MATES staff as honest, reliable, proactive and relationship based.** **Improved helping behaviours.** Increased individual and site resilience. Improved interpersonal relationships among MATES program participants and strengthened interpersonal connections (mateship) Workers find ways to extend MATES onsite. Evidence of active mental health alliance across all levels in the workplace A model framework implemented, demonstrating an industry –wide approach to mental health.	Psychological distress in the construction industry is reduced. **Suicide in the construction industry is reduced.** MATES values and culture are established across industry, increasing help seeking and social connection, reducing public stigma, mandating MATES in tenders and increasing compassion in workforce … that MATES is a ‘way of doing business’. Sites are running MATES on their own. Mental health plans are regulated across Australian workforces.

MATES Program Logic Model Outcomes

**Bold text** denotes that this review found relevant evaluation for the outcome.

Source (LaMontagne and Shann, 2020).

## METHODS

### Approach and eligibility criteria

The systematic review for this study followed the guidelines of the Preferred Reporting Items for Systematic Review and Meta-Analysis (PRISMA) (Liberati *et al.*, 2009). The search was conducted by Author 1 with reviewing and checking of the articles by co-authors. Co-authors included two persons who were authors of studies included in this review, plus a review co-author who was not an author or affiliated with the reviewed studies. For inclusion, studies initially had to be based on data directly relevant to the MATES program and be published in a peer-reviewed journal prior to the cut off of 31 January 2023.

### Search strategy and data extraction

A literature search was conducted using the Scopus and APA PsycInfo databases and the Google Scholar search engine. The MATES research resource hub was also reviewed (MATES in Construction, 2022). A Boolean search for the term *MATES in Construction* was conducted across the first three platforms while all available records were scanned on the MATES research hub. The search was conducted on 1 February 2023. The first author screened titles and abstracts for inclusion and completed data extraction. Data from included studies were collated in a data extraction table, which included author/s, publication year, country where study conducted, study design, sample, MATES program component assessed; exposure and outcome measures and analysis methods; and results.

### Data synthesis and analysis

A narrative synthesis was carried out, with a focus on documenting how these results aligned or did not align with the MATES program logic model framework (LaMontagne and Shann, 2020). The primary outcomes of interest in relation to the program logic model were impacts on mental health and suicide prevention literacy, decreased public stigma, active engagement of workers and volunteers in mental health and suicide prevention, increased helping behaviours and the industry’s views of MATES and its role in the industry. Meta-analysis was not feasible for this systematic review given the heterogeneity in the measures and methodologies used in the included studies.

## RESULTS

**Fig. 1:**
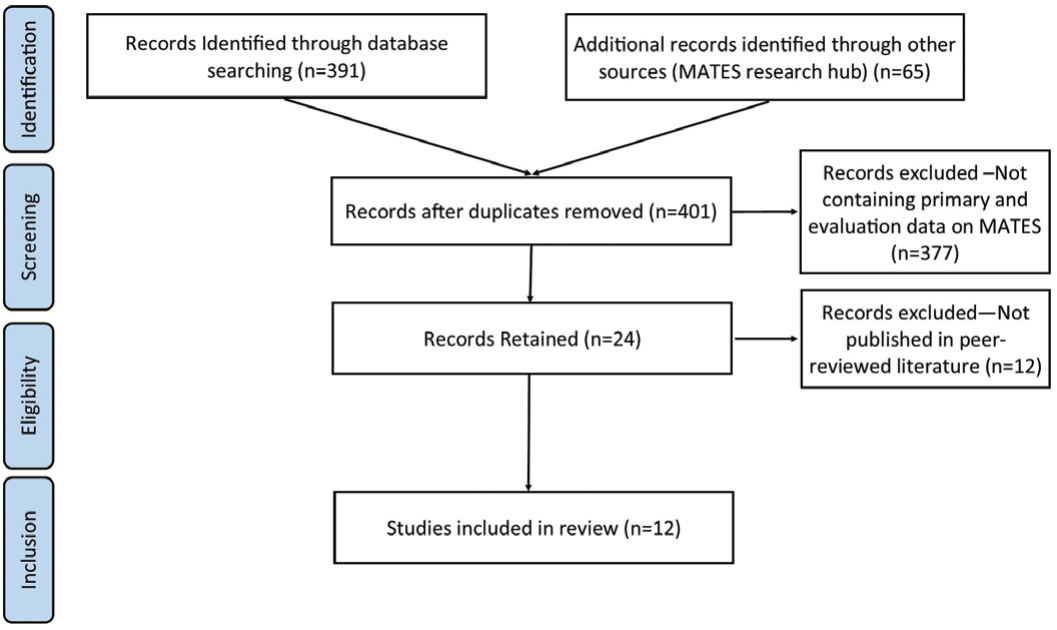
PRISMA diagram revision.

The searches and numbers of identified articles are summarized in Figure 1. The literature search identified a total of 456 records. After the removal of duplicates, 401 title and abstracts were screened. Of these, 24 studies were retained for full-text review whereof 12 were retained for inclusion in the systematic review, these are summarized in Table 2.

**Table 2:** Summary of included studies

Author	Study type	*N*	Program logic outcome	Program elements evaluated	Results
Gullestrup *et al.* (2011)	Cross-sectional: non-equivalent group comparison	7000	Short-term	GAT, Connector, Case Management	GAT participants (*n* = 7000) were asked five suicide awareness questions before and five after GAT training. The same questions were asked of a comparison *n* = 355 group not receiving GAT training. A Mann–Whitney *U* test was carried out on the full sample and on a randomly selected sample of *n* = 355 of the intervention group compared with the comparison group. There was no significant difference between the intervention and comparison group in the five questions asked before training (Mann–Whitney *p* = values between 0.06 and 0.85) while both the full sample and the randomly selected sample demonstrated significantly more desirable responses after training (Mann–Whitney *p* = values between 0.00 and 0.01). Mean responses varied from the intervention and comparison group between 1.8% higher agreement that mental health is a work health and safety issue, 8.1% higher agreement that suicide is everyone’s business and 11.7% higher agreement that most people with thoughts of suicide do not want to die. Connectors (*n* = 696) rated MATES very helpful (74.1%), agreed with the statement that ‘*MATES will work and could save lives on site*’ (74.1% Strongly agree, 24.9% agree), and with the statement ‘*I know where and how to get help now’* (74.6% Strongly agree, 24.2% agree), Case management and crisis line had positive engagement.
Doran *et al.* (2016)	Ecological: retrospective mortality study and health Economic Evaluation	N/A	Long-term	Overall program impact	A 10% reduction in relative risk (RRR = 0.9) of suicide behaviour amongst Queensland construction workers in the 5 years after MATES implementation compared with the 5 years prior. A potential return on investment by applying the program in NSW in the same manner as in Queensland was calculated at $4.6 per $1 invested.
Martin *et al.* (2016)	Ecological: retrospective mortality study	N/A	Long-term	Overall program impact	Age Standardized Suicide Rates (ASR) amongst Qld Construction workers fell from ASR = 28.9 (per 100,000) before MATES (2003–07) to ASR = 26.7 after (2008–12), representing a 7.9% shift, while Queensland’s suicide rate for all other employed males increased by 12.9% over the same period [ASR = 21.7 (2003–07) to ASR 24.5 (2008–12)]. The decrease was greatest amongst machine operators/labourers [ASR = 38.3 (2003–07) to ASR = 30.8 (2008–12), a decrease of 19.6%] while the ASR amongst skilled trades increased by 4.2% from ASR = 24.0 (2003–07) to ASR = 25.0 (2008–12). None of the changes achieved statistical significance.
Tynan *et al.* (2018)	Interventional pre–post	1277	Short-term	GAT, Connector	Significant pre- to post-training improvement in self-identified suicide prevention literacy and willingness to intervene and help a workmate following MATES in Mining GAT (*n* = 1163) and Connector (*n* = 114) training was found. Five statements about suicide prevention literacy and help offering confidence were asked and rated on a 5-point Likert scale. Ratings increased towards more desirable responses for GAT trained by between 7.4 and 13.0% and Connector trained between 15.6 and 19.2%—all at *p* ≤ 0.001. Connector participants had more desirable response at pre-training compared with GAT participants between 4.6 and 19.6% across the five statements. Training was rated on a scale of 1–5 as relevant (*M* = 4.4; SD = 0.7) and useful (*M* = 4.4; SD = 0.7), satisfactorily delivered (*M* = 4.5; SD = 0.6) and participants would recommend the training (*M* = 4.5; SD = 0.7).
King *et al.* (2018)	Interventional pre–post	20,125	Short-term	GAT	A sample of five pre- and post-GAT training question on suicide prevention literacy answered by *n* = 20,125 was analysed. Answers were on a 5-point Likert scale. A sub sample of *n* = 12,257 where occupation information was known was also analysed. Participants were also asked about helping behaviours. 75.1% had known someone who died by suicide, 39.1% had sought help for themselves while 77.7% had helped someone else who were struggling. On a score from 1 to 5 participants scored a mean 3.74 (SD = 0.92) on likelihood of seeking help in the future and a mean of 4.60 (SD = 0.57) on likelihood of offering help to a mate. The sample showed significantly more desirable responses post-training compared with pre-training. There was a 16% improvement to recognition that a person with thoughts of suicide may invite others to notice, an 8.5% improvement to recognizing mental health as a safety issue and an 8.8% improvement to seeing suicide as an industry issue (all *p* ≤ 0.001). Managers and Professionals had more desirable pre-training responses and larger post-training movement than lower-skilled workers such as Machinery Operators and Labourers (Recognizing invitations Managers = 21.3% vs Labourers = 13.8%, Suicide as a safety issue Managers = 11.5% vs Labourers = 6.4% and Suicide as an industry issue Professionals = 14.3% vs Labourers = 7.3%—all *p* ≤ 0.001).
King *et al.* (2019)	Interventional pre–post	19,917	Short-term	GAT	Five statements were graded on a five-point Likert scale by *n* = 19,917 participants. The sample was analysed according to age and skill level of participants. Young workers scored significantly less desirable responses to questions relating to recognizing that a person with thoughts of suicide will invite other to notice (7.5% less) and believing that talking about suicide could talk suicide compared with other workers (5.2% less). Young workers were more likely to see suicide as a work safety issue (2.9% more) while there was no significant difference based on age to the statement that suicide was an industry issue. Young workers had significantly larger shifts in acknowledging that a person may send out invitations (14.9% change among 15–24 year old vs 11.1% among 45+ years old) seeing suicide as a safety issue (3.8% for those aged 15–24 years vs 3.1% for those 45+ years) and seeing suicide as an industry responsibility (15–14 years old 4% vs 45+ years old 2.8%).
Ross *et al*. (2019)	Mixed method study	104	Short-term	Connector, Case Management, Overall impact	A sample of *n* = 104 Connectors were surveyed immediately prior to and after training. Further *n* = 10 individual interviews of case management clients and focus groups of *n* = 17 was conducted. A six-item survey requiring answers on a 5-point Likert scale was used to assess suicide literacy, help seeking and help offering intentions. A question about safety in discussion of suicide showed a 6.83% (*p* ≤ 0.001) shift toward a more desirable response post-training. There were also positive changes in recognition of workplace support for well-being improved 6.94% shift (*p* ≤ 0.001), while more there was pre–post-training increase in those intending to seek help if in crisis (6.99%, *p* ≤ 0.001). Three items inquired about help offering and also yielded positive changes—‘would you notice a mate struggling’ shifted by 16.67% (*p* ≤ 0.001), willingness to seek help improved by 2.3% (*p* = 0.01) and knowledge of how to help connect a mate to help improved by 19.6% (*p* ≤ 0.001). When rating where participants would seek support on a scale 1–5 an Intimate Partner (Mean = 4.3) was most supported followed by Close Family (Mean = 4.1), Friend (Mean = 4.0), MH Professional (Mean = 3.9), MATES Worker (Mean = 3.9), Doctor (Mean = 3.71), Workmate (Mean = 3.4), Helpline (Mean = 3.3) and the least supported was a supervisor (Mean = 0.3.0) and Minister/Religious leader (Mean = 1.8).
Sayer *et al.* (2019)	Interventional pre–post collected at baseline, 6- and 18-month follow-up	1651	Short-term	Overall program impact	Participants (*n* = 1651) at two mine sites were surveyed at three time points (pre-intervention, 6- and 18-month post-intervention). Three questions around perceptions of mental health stigma were asked relating to friends, workmates and employer were asked. There was significant improvement from pre- to 18 months on perception of stigma from friends (15.7% improvement, *p* ≤ 0.001) and from workmates (15.1% improvement, *p* = 0.01). There was some indication that stigma from the workplace improved (16.0%), noting the *p*-value = 0.07. Participants were also asked to rate their most likely sources for support for a mental health crisis at each of the three time points. Family (T1 = 75.6%, T3 = 82.4%, improvement 9.0%, *p* = 0.05), Friends (T1 = 72.4%, T3 = 81.5%, improvement 12.71%, *p* = 0.02) and General Practitioner (T1 = 65.2%, T3 = 68% improvement 4.26%, *p* = 0.1) were the sources that were most highly rated. The largest shift was observed for supervisors (T1 = 25.6%, T3 = 35.2% improvement 37.5%, *p* ≤ 0.001) noting that they remained lowest among all potential sources of support. Apart from supervisors, other resources improving significantly between T1 and T3 were EAP service (T1 = 30%, T3 = 49.5%, improvement 65% *p* ≤ 0.001), Colleagues (T1 = 33.7%, T3 = 45%, improvement 33.53% *p* ≤ 0.001) and MATES staff and volunteers (T1 = 39.2%, T3 = 46.8%, improvement 19.39% *p* = 0.12).
Ross *et al.* (2020a)	Interventional pre–post collected at baseline and 3-month follow-up	2977	Short-term	GAT, MAT	MAT participants (*n* = 717) and GAT participants (*n* = 2260) were recruited to participated in an immediate pre- and post-training survey and 3 months longitudinal follow-up. The study found no difference in results between MAT and GAT participants. The study found a significant time effect on suicide literacy at a 3-month follow-up. The only suicide literacy effect retained at follow-up was a respond to the statement ‘*If my mate was going through a difficult time feeling upset or thinking about suicide, I would know how to connect him/her to appropriate help’*. A mean difference of −0.38 (*p* ≤ *0.001)* was maintained from pre-training to 3-month follow-up. Relating to help-seeking intentions and resources, only two resources maintained improvement at 3-month follow-up being ‘*Mates worker/ connector*’ A mean difference of −0.59 (*p* ≤ 0.001) and Telephone Helpline (such as the MATES support line) at a mean difference of −0.36 (*p* = 0.01).
Ross *et al*. (2020b)	Interventional pre–post	4887	Short-term	GAT	Data were collected from GAT participants (*n* = 4887) in the MATES in Energy program. Participants were asked to rate 7 statements relating to suicide literacy and stigma on a five-point Likert scale. All statements demonstrated a statistically significant shift toward more desirable response post-training at the *p* ≤ 0.001 level. A statement relating to willingness to offer help had the smallest improvement of 2.6% but this could be due to a ceiling effect; shifts in other items were between 6.4 and 27.2%. Furthermore, participants were asked to identify where they would seek help from if in crisis (yes/no) before (*B*) and after (*A*) training with all resources showing significant improvement: Family (*B* = 61.9%, *A* = 66.89%, 8%, difference, *p* ≤ 0.001), Friend (*B* = 50.3%, *A* = 57.1%, 13.5% difference, *p* ≤ 0.001), Doctor (*B* = 46.1%, *A* = 48.9%, 6% difference, *p* = 0.01) Counsellor (*B* = 28.4%, *A* = 40.1%, 41.1% difference, *p* ≤ 0.001), Help Line (*B* = 19.4%, *A* = 39.02%, 101.4% difference, *p* ≤ 0.001), Workmate (*B* = 21.7%, *A* = 35.2%, 62.2% difference *p* ≤ 0.001), Psychologist (*B* = 28.9%, *A* = 34.7%, 20% difference, *p* ≤ 0.001) and Supervisor (*B* = 13.9%, *A* = 21.5%, 55% difference, *p* ≤ 0.001).
Doran *et al*. (2021)	Analysis of existing data, small exit survey	N/A	Short-term	Case Management	Demand for MATES case management had increased significantly between 2010 and 2018 (265%) with a confirmed upward trend over time (tau = 0.583, *p* ≤ 0.001). Most common occupations for workers case managed were Labourers (30%), plant operators (17%) and plumbers (14%). Most common presenting issues were relationships (38%), work-related concerns (27%) and family concerns (22%). Most clients were male (92%) with a median age of 39 years of age. Most common referrals from case management were to Employee Assistance Programs (48%), counselling and well-being services (12%) and medical professionals (5%). In 25% of cases, no referrals were noted.
Maheen *et al*. (2022)	Ecological: retrospective mortality study	N/A	Long-term	Overall program impact	Age-standardized suicide rates declined significantly more amongst construction workers compared with other employed men in Australia in the period between 2001 and 2019. Over the full period there were 3995 suicides amongst construction workers (ASR = 26.6/100,000, *p* ≤ 0.001) varying between ASR = 25.8 and ASR = 27.4, compared with 10,287 suicides amongst other employed men (ASR = 13.2/100,000, *p* ≤ 0.001) varying between ASR = 12.9 and ASR = 13.4. An average annual percentage change (AAPC) was calculated showing an annual decrease of 3% per year for Construction workers and 1.5% decrease for other employed men. The difference was statistically significant with a pair-wise comparison showing an AAPC of 1.4% (*p* ≤ 0.001).

N/A, not applicable.

### Overview of study designs and methods

Most studies (*n* = 9) were carried out among samples of construction workers (Gullestrup *et al.*, 2011; Doran *et al.*, 2016, 2021; Martin *et al.*, 2016; King *et al.*, 2018, 2019; Ross *et al.*, 2019, 2020a, Maheen *et al.*, 2022), two were in coal mining populations (Tynan *et al.*, 2018; Sayers *et al.*, 2019) and one was in the energy sector (Ross *et al.*, 2020b). Most (*n* = 7) used quantitative survey methods (Gullestrup *et al.*, 2011; King *et al.*, 2018, 2019; Tynan *et al.*, 2018; Sayers *et al.*, 2019; Ross *et al.*, 2020a, 2020b), while two were mixed method studies (Ross *et al.*, 2019; Doran *et al.*, 2021). Three articles were based solely on data from coroners’ death investigations (Doran *et al.*, 2016; Martin *et al.*, 2016; Maheen *et al.*, 2022). Six studies used immediate pre- to post-training designs (Gullestrup *et al.*, 2011; King *et al.*, 2018, 2019; Tynan *et al.*, 2018; Ross *et al.*, 2019, 2020b), one study used a longitudinal design (Ross *et al.*, 2020a), while one study used a repeat cross-sectional survey (Sayers *et al.*, 2019). One study included a comparison group (Gullestrup *et al.*, 2011) but there were no experimental studies (with random assignment to intervention or comparison/control). Findings were synthesized with reference to the relevant program logic model heading. A list of program logic model outcomes with the headings used in this article highlighted is found in Table 1.

### Program logic model identified short-term outcomes

1.‘Increased Mental Health and Suicide Literacy and Decreased Public Stigma’

#### GAT

Five studies identified statistically significant improvements in mental health and suicide prevention literacy from pre- to post-GAT (Gullestrup *et al.*, 2011; King *et al.*, 2018, 2019; Tynan *et al.*, 2018; Ross *et al.*, 2020b). Data collection for these studies was conducted immediately before and after the training. One study used a non-intervention group for comparison and found that while there was no difference in responses to five mental health and suicide prevention literacy questions pre-GAT between participants in training (*n* = 7311) and a comparison group (*n* = 355), there was a significant, favourable post-training improvement in responses to five questions that were not observed in the non-intervention group (*p* ≤ 0.01) suggesting a positive impact of the training on mental health and suicide prevention literacy and stigma (Gullestrup *et al.*, 2011). Five other large quantitative studies (*n* = 2977 up to *n* = 20,125), using similar pre–post-survey instruments, demonstrated improvement in suicide prevention literacy and decreased stigma in pre-GAT to immediately post-GAT testing (King *et al.*, 2018, 2019; Tynan *et al.*, 2018; Ross *et al.*, 2020a, 2020b).

One study (Ross *et al.*, 2020a) used a longitudinal design to examine the consistency in outcomes between two modalities of providing mental health and suicide prevention awareness training on sites: GAT for larger sites and MAT for smaller sites. The study collected data pre-training (T1), post-training (T2) and at 3 months on-line follow-up (T3). Acknowledging the limitation of substantial loss to follow-up (T1, *n* = 2977; T3, *n* = 245) the study found that there was no significant difference between the modality of delivery (GAT vs MAT). While there was a significant improvement in suicide literacy and stigma reduction from pre- to post-training, most of this effect was lost by the 3 months follow-up. However, help-offering intention, a core objective of the MATES program, did maintain a significant improvement from T1 to T3 (*p* ≤ 0.001).

Sayers et al. examined outcomes from a MATES intervention in two coal mines (Sayers *et al.*, 2019). A repeat cross-sectional survey was administered at three time points across both mines: pre-intervention (T1, *n* = 649), 6 months (T2, *n* = 608) and 18 months (T3, *n* = 394) post-GAT training. Participants showed significant improvements over the 18 months in attitudes to public- and self-stigma, disagreeing with a statement that they would be treated differently by friends (T1 = 68.3%; T2 = 68.9%; T3 = 79%; *p* ≤ 0.001) or colleagues (T1 = 62.1%; T2 = 63.9%; T3 = 71.5%; *p* = 0.01) if they knew about them having a mental health condition. The same was not observed for structural stigma [referring to policies, procedures and cultural norms that restrict the opportunities of those with mental illness (Reavley, 2021) where fewer workers disagreed with the statement that their workplace would not treat them differently if it knew about them having a mental health condition and no significant improvement was observed over time (T1 = 56.6%; T2 = 60.8%; T3 = 65.7%; *p* = 0.07).

#### Influence of socio-demographic characteristics on outcomes

The studies showed that the socio-demographic characteristics of participants modified the impact of training on outcomes. For example, one study of *n* = 12,853 participants found that white-collar workers reported better mental health and suicide prevention literacy than blue-collar workers and white-collar workers also had a greater improvement from pre- to post-training (*p* < 0.001) (King *et al.*, 2018). Two studies (King *et al.*, 2019; Ross *et al.*, 2020b) analysed the effect of age on worker responses to pre- and post-mental health and suicide prevention literacy questions. Young workers had less desirable responses to several mental health and suicide prevention literacy questions but also had a greater pre- to post-training improvement than their older colleagues (King *et al.*, 2019; Ross *et al.*, 2020b). One study showed gender differences in GAT responses amongst energy industry workers (Ross *et al.*, 2020b). At pre-training women provided significantly more desirable responses to help-seeking intentions (*p* < 0.001) while men had a significantly greater improvement from pre- to post-training responses (*p* = 0.01) (Ross *et al.*, 2020b).

#### Influence of lived experience on outcomes

Lived experience of suicide also appeared to influence the impact of GAT outcomes (Ross *et al.*, 2020b). A study of *n* = 4788 energy workers found that 65% knew someone who died by suicide, 70% had known someone who attempted suicide and 2% reported current or recent suicide ideation. People without lived experience of suicide loss had a more desirable change in responses relating to safety in talking about suicide (*p* = 0.04) while those who did not have lived experience of a suicide attempt had a more desirable change in responses recognizing the industry’s role in suicide prevention (*p* = 0.001) (Ross *et al.*, 2020b). Lived experience of suicide ideation did not appear to impact pre- to post-training outcomes (Ross *et al.*, 2020b).

2. ‘Workers and Volunteers Play an Active Role in Better Mental Health and Suicide Prevention’

Two quantitative studies focussed on the roles and experiences of MATES volunteers on sites (Gullestrup *et al.*, 2011; Ross *et al.*, 2019). One study (Gullestrup *et al.*, 2011) considered responses following Connector training (*n* = 604). After training participants agreed that they could save lives in their workplace (99%), knew where to get help and support (99%) and they felt prepared to have discussions about suicide (98%). Important for program diffusion, 99% of participants intended to tell someone about the MATES program (Gullestrup *et al.*, 2011). A second study (Ross *et al.*, 2019) conducted a pre- and post-survey on the training of 104 Connectors. Participants showed significant improved suicide awareness, help-seeking and help-offering intentions from pre- to post-training (all *p* ≤ 0.001). The emotional well-being of the participants was also improved from pre- to post-training (Ross *et al.*, 2019).

A qualitative analysis (Ross *et al.*, 2019) of focus groups conducted with *n* = 17 Connectors highlighted several key themes important to understanding the role of Connectors, including findings of relevance to the MATES model of Outrage, Hope and Action. Many volunteers were motivated by the very high suicide rates in the industry (Outrage) and felt the Connector training provided them with the right awareness and skills to be confident in the role (Hope and Action). Connectors felt that it was important that MATES was an inherent part of *their industry* and *their workplace*. They found the MATES model simple to engage with and created a movement for suicide prevention by building both passion and camaraderie.

3. ‘Workers and/or Family Members Obtain Support from MATES or Volunteers’

#### Help seeking and help offering

Four studies (Ross *et al.*, 2019, 2020a, 2020b; Sayers *et al.*, 2019) analysed workers’ views on the most suitable sources for help and support. In these studies, workers were generally asked about the likelihood of seeking help or using the resources to offer help, from a pre-nominated list. Across all studies, workers were most likely to nominate informal resources such as family, friends and workmates as most useful to them. MATES-related resources such as Connectors, ASIST (Applied Suicide Intervention Skills Trained) workers, Field Officers or helplines were also likely support resources. All four studies, conducted across the construction (Ross *et al.*, 2019, 2020a), mining (Sayers *et al.*, 2019) and energy (Ross *et al.*, 2020b) industries found supervisors as the least preferred resource for help and support.

MATES training led to a significant increase in intention or likelihood of help seeking and help offering from all nominated sources from pre- to post-training (all *p* ≤ 0.01). One study also found that this improvement was maintained for the MATES-related resources, MATES worker/Connector (*p* < 0.001) and Helpline (*p* = 0.01) longitudinally over 3 months (Ross *et al.*, 2020a).

Over an 18-month period post-training, a study in two coal mines showed improvements in help-seeking intentions maintained after 18 months for intentions to seek help from supervisors (*p* < 0.01), family members (*p* = 0.05), friends (*p* = 0.02), colleagues (*p* < 0.01), employee assistance programs (*p* < 0.01) and psychologists (*p* = 0.04) (Sayers *et al.*, 2019).

#### Case management support

Three studies analysed the MATES case management services (Gullestrup *et al.*, 2011; Ross *et al.*, 2019; Doran *et al.*, 2021). Approximately 7% of MATES participants accessed case management over a 2½ year period (Gullestrup *et al.*, 2011). A time trend analysis showed the need for case management increased with the on-site program expansion with a 265% increase in demands between 2010 and 2018 (*p* < 0.001) (Doran *et al.*, 2021). The uptake of case management support across gender and age groups were consistent with that observed amongst MATES training participants generally (King *et al.*, 2018; Ross *et al.*, 2020a). Lower-skilled trades were overrepresented as case management clients. Labourers made up 30% of clients and 25% of training participants, machine operators 17% of clients against 14% of training participants. Managers were underrepresented in case management 11% of clients against 17% of training participants (King *et al.*, 2018).

#### Experience of case management clients

A qualitative analysis of MATES case management client experiences was conducted through interviews with eight construction workers and two partners of clients (Ross *et al.*, 2019). Case management users reported that cultural factors, specifically the masculine culture of the industry including the importance placed on being the ‘provider’ and stoicism were barriers to engaging in support. Participants explained how the MATES program overcame this by being highly visible, promoted and embedded in the construction industry. It was important that the MATES program used industry-specific language and imagery, making people feel safe and confident in connecting with MATES. Having peers and industry leaders such as union delegates sharing their lived experiences of help seeking and help offering was also very important to overcoming the barriers to accessing support (Ross *et al.*, 2019).

### Program logic model identified medium-term outcomes

1.‘Industry see MATES staff as honest, reliable, proactive, and relationship based’

A qualitative study of Connectors highlighted the passion and engagement of the MATES staff with industry as integral to the success of MATES. The study identified a ‘huge camaraderie’ around the program and field staff as very popular with workers on site. This was also reflected in interviews with case management clients pointing to the importance of the peer workforce creating a feeling of MATES being part of the construction industry (Ross *et al.*, 2019).

2.‘Improved Helping Behaviours’

The program logic model presumes workers play an active role in protecting and promoting mental health leading to improved helping behaviours generally. Two studies analysed referral sources for MATES case management showing a shift from help seeking towards help offering, as demonstrated by referrals changing over time from 44% of clients self-referring in 2011 falling to 22% in 2021. Referrals initiated by MATES staff and volunteers (referring to Connectors and ASIST workers) increased from 11% in 2011 to 22% in 2021, and referrals from unions increased from 14% in 2011 to 20% in 2021 (Gullestrup *et al.*, 2011; Doran *et al.*, 2021).

### Program logic model identified long-term outcomes

1.‘Suicide in the Construction Industry is Reduced’

Three studies (Doran *et al.*, 2016; Martin *et al.*, 2016; Maheen *et al.*, 2022) analysed suicide risk and suicide rates in the construction industry over time all based on Australian National Coronial Information System and Australian Bureau of Statistics (ABS) labour force data. Two studies analysed suicide rates in the Queensland construction industry (Doran *et al.*, 2016; Martin *et al.*, 2016), and one across the Australian construction industry (Maheen *et al.*, 2022).

One study found a non-significant reduction in suicide rates in Queensland 5 years after the introduction of the MATES program of 7.9% (T1 = 28.9/100,000; T2 = 26.7/100.000; *p* = 0.386) against a slight increase for other employed males (Martin *et al.*, 2016). Another study (Doran *et al.*, 2016) also analysing suicide rates amongst Queensland Construction workers in the 5 years pre- and post-MATES calculated a relative risk ratio of RRR = 1:0.9 suggesting a reduced risk of suicide for Queensland construction workers of 9.6% (95% CI = 9.1–10.0%) between the two periods.

A recent study (Maheen *et al.*, 2022) analysed suicide rates amongst Australian male construction workers compared with other employed men in Australia between 2001 and 2019 and calculated an annual average percentage change in suicide rates of −3% per year for construction workers in comparison to −1.5% amongst other employed men (*p* < 0.001). The authors noted these national trends would most likely be attributable to a range of general population initiatives over this period but also construction-specific initiatives including the MATES program.

## DISCUSSION

This review found evidence supporting the achievement of some outcomes identified in the MATES program logic model. Evidence was found of a positive impact on suicide prevention and mental health literacy. Some, albeit limited evidence was found of a positive impact on mental health stigma and helping behaviours over an 18-month period. Support was also found for the role of peer-focussed activities on worker empowerment and ownership of the program as important to both help offerors and help seekers. Studies examining help-seeking intentions suggested that workers had a strong preference for relational help-seeking resources (friends, family, colleagues) over employer-structured resources such as Employee Assistance Programs and supervisors or professional mental health resources. Findings also suggest that MATES led to increased help-seeking intentions from MATES-specific resources such as Connectors and the MATES helpline and that this increase was maintained over time. These findings provide some, although limited, support for the effectiveness of the MATES ‘Outrage, Hope, Action’ Model in engaging construction workers in suicide prevention. Finally, studies found some evidence that MATES has had a positive impact on suicide rates and relative suicide risk in the construction industry.

Previous workplace suicide prevention systematic reviews have focussed on specific industries (Bagley *et al.*, 2010; Witt *et al.*, 2017) or the field of workplace suicide prevention generally across different intervention programs (Takada and Shima, 2010; Milner *et al.*, 2015). In terms of contextualizing these results in the wider body of research, our results broadly align with other workplace suicide reviews, as detailed below.

Previous reviews of workplace programs described in the peer reviewed and grey literature found that very few programs were well articulated in the literature and even fewer evaluated (Milner *et al.*, 2015). Takada and Shima studied characteristics and effects of suicide prevention programs in workplace and other settings and found a lack of coherent strategy linking individual program elements in many suicide prevention programs (Takada and Shima, 2010). The MATES program logic model articulates the intended links between elements, enabling the program to be assessed systematically (LaMontagne and Shann, 2020). The present review is, to our knowledge, the first systematic review of the published evidence of a single multimodal workplace suicide prevention program. This narrow focus allows for a deeper understanding of how individual program components interact or impact on the overall program outcome (Takada and Shima, 2010; Milner *et al.*, 2015), and the programs potential impacts on suicide rates in the construction industry (Bagley *et al.*, 2010).

Witt *et al*. identified 13 studies evaluating suicide intervention programs targeting protective or emergency services (Witt *et al*., 2017). Despite finding a halving of suicide rates in the included studies, a limitation identified in the study was the inability to ascribe causality in community-wide multicomponent interventions. Bagley et al. in their review of suicide prevention programs amongst military or veterans identified that multicomponent programs including education, gatekeepers, screening for individual risk as well as reduced access to means of suicide and improved access to mental health support were associated with reduced suicide rates (Bagley *et al.*, 2010). In line with these two reviews, the present review also identified significant reductions in suicide rates amongst construction workers, double that observed amongst other employed men as well as a reduced risk of suicide within the industry (Doran *et al.*, 2016; Martin *et al.*, 2016; Maheen *et al.*, 2022). Like previous reviews, this study was unable to draw a causal link between these reduced rates and the intervention. Previous studies have suggested an association between large-scale multimodal workplace suicide prevention programs and other public health benefits including reduced homicide, accidental deaths and family violence (Knox *et al.*, 2003, 2010). This is an area for further study in the MATES program context.

Witt et al. identified that most workplace programs focussed on secondary and tertiary-level prevention activities with only a few programs considering work environment factors (Witt *et al.*, 2017). The MATES program logic model described change to environmental factors as a long-term outcome of the program establishing MATES values and culture across the industry, making ‘MATES a way of doing business’ (LaMontagne and Shann, 2020). While this review did not find any published evidence for such a cultural shift occurring, the grey literature described a collaboration between MATES, the industry and researchers to lead cultural and environmental change in the industry. Examples of such initiatives include an industry blueprint for better mental health and suicide prevention (Milner 2017; MATES in Construction, 2018), initiatives focussing on bullying, mental health and suicidality amongst apprentices (Ross *et al.*, 2020c, 2021, 2022) and understanding distress amongst construction workers (Meurk, 2021). This aspect of the MATES program also requires further development and evaluation.

Worker engagement and collective action are central to the MATES program (LaMontagne and Shann, 2020). We found some support for the MATES ‘Outrage, Hope, Action Model’ of engagement in several qualitative studies (Ross, 2017; Ross *et al.*, 2019). This model is consistent with the Social Identity Model for Collective Action (Van Zomeren *et al.*, 2008) describing it as a model for mobilizing people to participate in social protest. This approach is novel in suicide prevention and may have wider application in overcoming difficulties with diffusion of preventative public health innovations (Rogers, 2002). Further research into this aspect of the MATES program may have wider implication for public health promotion in male-dominated cultures generally.

A limitation of the available evidence of the MATES program is the lack of analysis of the role of masculine norms. The impact of masculinity on suicide risk and in suicide prevention is complex and cannot be explained by lack of help seeking alone (Chandler, 2022). The gender paradox of suicide necessitate to ask not only how masculinity impacts higher suicide rates amongst men but also how it impact lower rates of suicide attempts (Canetto and Sakinofsky, 1998; Keohane and Richardson, 2018; Milner *et al.*, 2020). The MATES program draws on Kiselica and Englar-Carlson’s (Kiselica and Englar-Carlson’s, 2010) strength-based Positive Psychology/Positive Masculinity model with focus on help offering and peer support between men over traditional a help seeking. Further research is needed on the role of masculinity in the MATES program context.

This study highlights the complexity of the MATES program logic model, and that further research is required to fully assess the effectiveness of the MATES program. The published evidence to date was limited by the lack of experimental designs as well as a deep qualitative documentation of how the program functions on worksites. For future research, a focus on medium-term outcomes such as improvement to individual and site resilience, reduction in stigmatizing behaviours, improved interpersonal relationships, worker ownership and extensions to the MATES program on sites and the creations of cross-industry alliances for better mental health and suicide prevention is required. From a broad public health perspective, it is significant that very limited evidence exists on the effectiveness of the MATES ‘Outrage, Hope, Action’ Model for diffusion of the MATES program.

### Strengths and limitations

This review has a number of strengths. It demonstrates the utility of an articulated MATES program logic model in framing and interpreting evaluation findings, and that further research is required to fully assess the impacts of the MATES program. It has applied a systematic approach following PRISMA guidelines to maximize transparency and reproducibility. Included studies generally had very high participation rates as data were collected as an integrated part of delivering the program, in particular the training elements of the program which target a minimum of 80% of all workers on site. This is the first review of the peer-reviewed evaluation research on the MATES program, providing a relatively detailed portrayal of a single program, complementing other reviews combining findings across workplace programs.

This review has several limitations. While restriction to peer-reviewed literature optimized the scientific quality of the included papers, this approach may also create a risk of publication bias. The variety of measures used in included studies precluded a meta-analysis, thus requiring a narrative synthesis of findings. We also note limitations in terms of generalizability, given that all included studies were conducted in Australia.

## CONCLUSIONS

While the MATES program is well documented in the literature and has a published program logic model, evaluation research on the MATES program to date has focussed on near- to medium-term outcomes, often with low causal inference research designs. While the current evidence is favourable, future research should prioritize higher causal inference studies and more emphasis on longer-term outcomes. From a broader public health perspective, further evaluation of the implementation and effectiveness of the MATES ‘Outrage, Hope, Action’ engagement model may inform strategies for the diffusion of MATES and other suicide prevention programs.

## Supplementary Material

daad082_suppl_Supplementary_Material
