# Supplementary material for: Effectiveness of the Australian MATES in Construction Suicide Prevention Program: a systematic review
Source: Health Promot Int. 2023 Aug 30;38(4):daad082. doi: 10.1093/heapro/daad082 (PMC10468011; doi:10.1093/heapro/daad082)
Supplement: daad082_suppl_Supplementary_Material [file daad082_suppl_supplementary_material.docx]

**Appendix**

**The MATES Program Explained**

MATES was set up in Queensland in 2007 as a bi-partisan initiative (unions and employer organisations) in response to research showing higher rates of suicide amongst Queensland construction workers (AISRAP, 2006, Heller et al., 2007, Neis and Neil, 2020). Since 2007 the program has extended across Australia and New Zealand and to other industries such as Mining, Energy and Manufacturing.

The core of the MATES program is a community development model around three levels of engagement. The three level of engagement are each designated with a hard hat sticker that makes it easy to recognise who is in the MATES program and which role they play. The roles are; **GAT** (General Awareness Training), **Connectors** and **ASIST** workers. GAT use an Anger, Hope, Action model to motivate workers to be engaged in suicide prevention in their industry. From GAT volunteer Connectors and ASIST workers are recruited. An important part of MATES is that workers are recruited as volunteers with the MATES program and therefore take their industry role with them as they change employers across the industry. The benefit of this focus is that the MATES program becomes a community movement and workers introduce the program to new sites and employers creating a viral effect. This volunteer network and training on sites is supported by MATES employed Field Officers.

As a suicide intervention model on a site the three levels of training establish a network of safety on site. GAT trained workers are aware of suicide as an issue and have basic skills to identify a co-worker in psychological distress. Connectors are alert to suicide risk, have the basic skills to start a conversation about suicide and to motivate a workmate to accept support. Connectors act as a connection point between GAT trained worker and suicide safety resources. ASIST workers are trained in applied suicide intervention skills and will help workers in distress and act as a suicide safety resource for Connectors on sites. The MATES supports these networks on sites by providing a 24/7 support line and case management support. The elements of the MATES program are set out in the table below.

MATES continually partners with researchers to develop and extend the program upstream from an intervention towards a prevention focus. Hence the MATES program is not static but continuously evolving toward a long-term object of a suicide safe construction industry. Examples of these extension are the development of the Australian Construction Industry Blueprint for Better Mental Health and Suicide Prevention, the Respond program developed to provide postvention and critical incident management skills to workers, the Supervisor Training program to develop and extend supervisors skills in meeting their obligations to provide a mentally safe workplace and a program targeting the mental health of apprentices.

| **MATES Program Components (Gullestrup et al., 2011, Ross et al., 2020)** | |
| --- | --- |
| **MATES Awareness Training** (MAT) | A conversational 15-minute toolbox talk for small groups of workers. MATs are conducted on small worksites often over the bonnet of a vehicle for groups of up to 15 workers. |
| **General Awareness Training** (GAT) | GAT trained workers are recognised by a white hard hat sticker. GAT is a one-hour suicide literacy educational session aimed at all workers onsite aimed at motivating workers for further engagement with the MATES program. GAT is designed for groups of 15 – 300 workers delivered on site. |
| **Connector** | Connectors are recognised by a green hard hat sticker. Connector training is a four-hour suicide alertness program incorporating LivingWorks safeTALK. Connectors are trained to connect workers in distress with further support. Connectors also act as advocates for the MATES program as they move from employer to employer. The target for Connector training is a minimum of 1 in 20 workers on site. The training is most often delivered on site. |
| **ASIST Worker** | ASIST workers are recognised by a blue hard hat sticker. ASIST worker training is a two-day LivingWorks Applied Suicide Intervention Skills Training workshop. Intervention training focus on creating as safety plan with people and help them make an assessment of what additional help is needed. ASIST is training is done in groups of up to 30 workers, most often done off site. |
| **Connector Meetings** | Connectors and ASIST workers on sites are encouraged to meet and discuss how the program is implemented and how they can support each other. |
| **Site Accreditation** | A site that has trained all workers in GAT, trained a minimum of 1 in 20 workers as Connectors, and trained at least one staff member in ASIST are eligible to become a MATES Accredited site. |
| **Site Activities** | MATES produce a range of material and activities that sites can engage with locally. Most noticeable is the annual Fly the Flag Day (coinciding with World Suicide Prevention Day) where sites locally organise and conduct activities celebrating the industry’s engagement in suicide prevention. |
| **Field Officer** | MATES employees who support program rollout on sites. FOs conduct training and help volunteer Connectors and ASIST workers perform their roles on site. Each participating site is allocated to a Field Officer responsible for building relationships and supporting the site. Field Officers also supports the volunteer network of Connectors and ASIST workers as they move across the industry from employer to employer. |
| **Case Manager** | A MATES employee employed to support individuals identified as experiencing a mental health crisis. Using a brokage model, case managers connect workers to support services. |
| **Support Line** | A 24/7 support line is available to all workers in the industry and their families. |
| **Research Reference Group** | A group of leading academics and researchers that advise and support MATES in commissioning and conducting quality research with the aim of evaluating and further developing the MATES program. |

Table 1 MATES program components

References:

AISRAP 2006. Suicide in Queensland's Commercial Building and Construction Industry. Brisbane: Australian Institute for Suicide Research and Prevention.

GULLESTRUP, J., LEQUERTIER, B. & MARTIN, G. 2011. MATES in construction: impact of a multimodal, community-based program for suicide prevention in the construction industry. Int J Environ Res Public Health, 8, 4180-96.

HELLER, T. S., HAWGOOD, J. L. & DE LEO, D. 2007. Correlates of Suicide in Building Industry Workers. Archives of Suicide Research, 11, 105-117.

NEIS, B. & NEIL, K. 2020. Mental health in the construction industry: an interview with Australia’s MATES in construction CEO, Jorgen Gullestrup. Labour & Industry: a journal of the social and economic relations of work, 1-17.

ROSS, V., CATON, N., GULLESTRUP, J. & KÕLVES, K. 2020. A Longitudinal Assessment of Two Suicide Prevention Training Programs for the Construction Industry. Int J Environ Res Public Health, 17.
